# Effects of Zwitterions on Structural Anomalies in Ionic Liquid Glasses Studied by EPR

**DOI:** 10.3390/nano13152164

**Published:** 2023-07-26

**Authors:** Olga D. Bakulina, Mikhail Yu. Ivanov, Sergey A. Prikhod’ko, Nicolay Yu. Adonin, Matvey V. Fedin

**Affiliations:** 1 International Tomography Center SB RAS, Institutskaya Street 3a, 630090 Novosibirsk, Russia; o.bakulina@tomo.nsc.ru; 2 Physics Department, Novosibirsk State University, Pirogova Street 2, 630090 Novosibirsk, Russia; 3 Boreskov Institute of Catalysis SB RAS, Lavrentiev Avenue 5, 630090 Novosibirsk, Russia; spri@catalysis.ru (S.A.P.); adonin@catalysis.ru (N.Y.A.)

**Keywords:** ionic liquids, zwitterionic liquids, librations, heterogeneities, nanostrucruting, EPR spectroscopy

## Abstract

Ionic liquids (ILs) form a variety of nanostructures due to their amphiphilic nature. Recently, unusual structural phenomena have been found in glassy ILs near their glass transition temperatures; however, in all studied cases, IL cations and anions were in the form of separate moieties. In this work, we investigate for the first time such structural anomalies in zwitterionic IL glasses (ZILs), where the cation and anion are bound in a single molecule. Such binding reasonably restricts mutual diffusion of cations and anions, leading to modification of nano-ordering and character of structural anomalies in these glassy nanomaterials, as has been investigated using Electron Paramagnetic Resonance (EPR) spectroscopy. In particular, the occurrence of structural anomalies in ZIL glasses was revealed, and their characteristic temperatures were found to be higher compared to common ILs of a similar structure. Altogether, this work broadens the scope of structural anomalies in ionic liquid glasses and indicates new routes to tune their properties.

## 1. Introduction

Ionic liquids (ILs) are a promising class of organic salts that are liquid at temperatures below 100 °C [1,2,3,4,5,6,7,8,9] and exhibit specific molecular self-organization and physicochemical properties, such as high thermal stability, low vapor pressure [8,10,11], and nano- and micro-heterogeneity [12,13,14]. These unique physical and chemical properties allow ILs to be used as new low-toxicity solvents in green chemistry [8,15,16], as drug delivery agents in biomedicine [17,18,19], and as components of new functional nanomaterials [20,21,22,23].

Generally, ILs possess high ionic conductivity due to the high mobility of their ions. However, in some systems, the conductivity of only one type of ion is required [24]. For instance, in lithium–sulfur batteries (LSB), it is necessary to avoid a polysulfide transfer (the so-called shuffle effect) to increase the lifetime of the battery [25,26,27]. Anions and cations in zwitterionic liquids (ZILs) are combined in one molecule; therefore, these ions have lower molecular mobility in comparison with classical ILs [28] and their migration and rearrangement in the bulk liquid are more restricted [29]. ZILs are able to demonstrate higher Li^+^ conductivity and higher suppression of polysulfide diffusion, which makes them interesting components of electrolytes [28].

Pure ZILs possess low ionic conductivity; however, they have been found to be excellent non-volatile solvents for various salts [29]. For instance, an addition of ZIL TLTFSI (tris(dioxa-3,6-heptyl)aminesulfonate coupled with bis(trifluoromethane)sulfonimide lithium salt) to LiNO_3_ (in percentage 30 vol.%/70 vol.%, respectively) allowed them to achieve higher nominal capacity and better capacity conservation after 500 cycles [28]. Yoshizawa et al. [24] demonstrated that the T_g_ of electrolytes influences ionic conductivity. T_g_ and ionic conductivity of the mixture are approximately constant as long as the ZIL’s mole fraction is less than 50%. At higher ZIL fractions, T_g_ starts to increase and ionic conductivity to decrease. Wu et al. compared structural peculiarities of ZILs and homologous ILs and demonstrated that free ether chains are segregated into nanodomains [30]. In addition, a significant intermediate ordering was found for ZILs, which is not observed for usual ILs. Moreover, interplay of electrostatic interactions between oppositely charged fragments of adjacent ZIL molecules and hydrophobic interactions between alkyl/vinyl/aryl substituents leads to formation of nanoaggregates [31]. Depending on the chemical structure of ZILs and the pH of the solution, the shapes of these nanoaggregates vary from spheres to flower-like aggregates, dendrites, etc.

Implementation of ILs in processing of cellulose and cellulose-based materials is another focus of recent research [32,33,34]. Classical ILs possess quite high cytotoxicity and viscosity, and are rather expensive [35]. This drawback can be remedied using zwitterionic liquids [36,37,38]. It has been demonstrated that zwitterionic structure is a crucial factor in the development of solvents with low toxicity for celluloses, because their zwitterionic nature hinders the hydrophobic interactions between the imidazolium N-alkyl chains and the phospholipids [38]. Thus, ZILs represent an interesting direction in current studies of ILs, and their potential applications are especially promising.

A recent series of IL studies using pulse electron paramagnetic resonance (EPR) spectroscopy revealed peculiar molecular dynamics in the glassy state near the glass transition temperature (*T*_g_) [39,40,41,42,43,44,45,46]. Such “structural anomaly” was first found in imidazolium-based ILs, and was later observed for other ILs, phthalates, alkyl alcohols, and alkyl benzenes [44]. Particularly, it was shown that the mobility of molecules decreases upon temperature increase in the range of 50–60 K below *T*_g_, and this phenomenon was attributed to specific structural rearrangements of ILs on the nanoscale. During the past 5 years, our group has scrupulously studied many aspects of this truly unusual phenomenon. First of all, we have reliably shown that the dynamics of alkyl chains, included in the IL structure, play a crucial role in the anomaly, which is fundamentally important [41]. Next, the influence of the spin-probe structure was excluded [43], and the anomaly was observed in glasses of other alkyl-chain-containing molecules (ionic liquids, phthalates, alkyl alcohols, and alkyl benzenes), thus making this phenomenon rather ubiquitous [44]. Furthermore, we studied the influence of water content in IL and found self-assembled domains around solutes in binary IL/water mixtures [47]. Using metal–organic frameworks as crystalline nanocontainers, we studied the influence of space restrictions on the occurrence and character of structural anomalies [42]. Finally, we studied the peculiarities of nanostructuring in Active Pharmaceutical Ingredient–Ionic Liquids [45].

Thus, many various structural factors have been varied to investigate the origin and specifics of structural anomalies in glassy ILs. However, in all cases, cations and anions acted as separate molecules, having a large degree of freedom to migrate and rearrange into polar/apolar domains in the bulk IL. In the present study, we investigated nanostructuring in zwitterionic liquids, where cations and anions were united in a single molecule. For this sake, the SO_3_^−^ group was attached to the alkyl chain of the cation, and such ZILs were studied along with their structural analogues—common ILs with separate [CH_3_SO_3_]^−^ anions. Below, we describe experimental observations for ZILs and structurally similar ILs, compare manifestations of structural anomalies, and conclude on the observed differences and future perspectives.

## 2. Materials and Methods

**Scheme 1:**
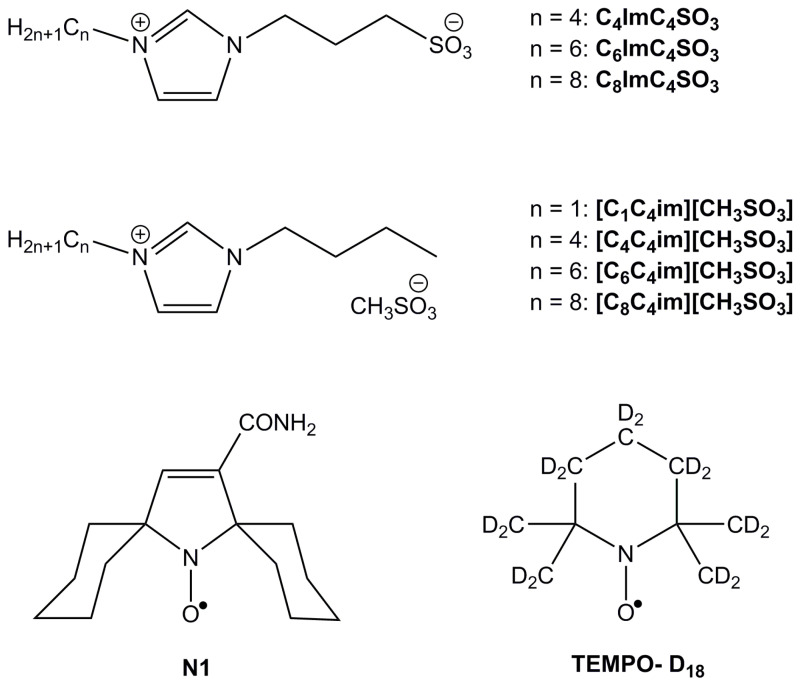
The chemical structures of the studied ILs and spin probes, N1 and TEMPO-D_18_.

**Materials and synthesis**. Scheme 1 shows the chemical structures of the ionic liquids 1-alkyl-3-methylimidazolium methanesulphonate ([C_n_mim][CH_3_SO_3_]), zwitterionic liquids 1-alkylimidazolium-3-butansulfonates (C_n_C_4_imSO_3_), and spin probes N1 and TEMPO-D_18_ used in this study. To investigate the influence of cationic alkyl chain mobility on the nanostructure of organic glasses we prepared a set of probe solutions in ILs and ZILs with n = 4, 6, and 8 (see Supplementary Materials for experimental details).

**EPR spectroscopy**. EPR measurements were carried out on a commercial Bruker Elexsys E580 spectrometer at X-band (9 GHz). The spectrometer was equipped with an Oxford Instruments temperature-control system (4–300 K) including a flow-through helium cryostat and a temperature controller. The echo-detected EPR spectra and phase memory times were recorded using the standard two-pulse Hahn echo sequence (pulse lengths were 100 ns for π and 50 ns for π/2). Theoretical simulations of the spectra were carried out using the EasySpin software package (v. 5.2.35) in the Matlab environment [48].

**Methodology**. Incorporation of paramagnetic probes into naturally diamagnetic media for EPR detection has often been used previously in IL research [49,50,51,52,53,54,55]. Following this strategy, we dissolved the radical probe in each studied IL at a concentration of ca. 1 mM and placed the solution into an EPR quartz tube with a 3.8 mm outer diameter. Then, the sample was evacuated at 10^−5^ mbar for 2 h to reduce the amount of remaining water and to eliminate the remaining oxygen. Finally, the sample was exposed to 3–5 freeze–pump–thaw cycles and sealed off under vacuum.

In all experiments, the sample was shock-frozen in liquid nitrogen and then transferred into the cryostat. This was done to guarantee that the sample was in the glassy state at the start of each experiment. The heating/cooling rates were ca. 1 K/min; the sample was kept in the cryostat for at least 10 min prior to each EPR measurement to reach equilibrium temperature.

To observe and investigate structural anomalies in IL glasses, we employed the combination of continuous wave (CW) and pulse EPR techniques, as was used in a series of our previous works [39,40,41,42,43,44,45,46,47,56]. The pulse EPR study relies on the methodology of stochastic molecular librations, previously developed and broadly applied by Dzuba et.al. to study molecular mobility in biological samples [57,58,59]. Namely, a robust approach to characterize the librations of nitroxide probes in molecular glasses and biopolymers using the pulse EPR of the embedded nitroxide probe has been developed. Stochastic librations of molecules are typical for glasses and refer to the small-angle wobbling of molecules driven by thermal energy. When the spin probe is dissolved in the glassy media, it follows the librations occurring in the surrounding matrix. In other words, being embedded in the glass, the spin probe tracks its molecular mobility. Thus, spin probes in the glasses are good reporters of their physical properties. To study the rigidity/density of the local nanoenvironment of the spin probe, a libration parameter *L* is used. In the case of nitroxide radicals, the transverse relaxation time *T*_2_ is different at different positions of the EPR spectrum due to the anisotropy of the hyperfine interaction between the unpaired electron and ^14^N nucleus (see Supplementary Materials for more details). It has been demonstrated that the difference between relaxation rates obtained in two spectral positions (with the maximum anisotropy *T*_2_(II) and the minimum anisotropy *T*_2_(I)) is connected with the motional parameter 〈α^2^〉τ_c_ as follows: *L* ≈ (1/*T*_2_(II) − 1/*T*_2_ (I)) = 10^11^〈α^2^〉τ_c_, where 〈α^2^〉 is the mean-squared amplitude of librations and τ_c_ is their characteristic time [57,58,60].

CW EPR allows the obtaining of complementary information on the local environment of the spin probe dissolved in the studied media. It has been shown that the observed spectrum is a superposition of two spectra of spin probe described by different models of motion. The first refers to the diffusive rotation of mobile radicals, whereas the second one describes immobilized nitroxide radicals. To simulate the spectral evolution vs. temperature, we used linear combinations of the above spectra with the weights (1 − M) and M for the immobile and mobile components, respectively.

**Quantum chemical calculations.** For the species C_n_C_4_ImSO_3_, [C_n_C_4_im]^+^ (n = 4, 6, and 8) and [CH_3_SO_3_]^−^, Van der Waals volumes were calculated using “atoms in molecules” (AIM) partitioning of electron density distributions [61]. The geometries of the selected ions and zwitterions were optimized using DFT-B3LYP/def2-TZVP calculations in the Orca software (v.5.0.4) [62]. The Bader analysis was performed using the Multiwfn program [63] where Van der Waals volumes were obtained (coordinates of atoms and volumes of selected species are given in the Supplementary Materials).

## 3. Results and Discussion

We selected a set of zwitter ILs, all of which have an imidazolium-based cation [C_n_C_4_im]^+^ (n = 4, 6, and 8) and [SO_3_]^−^ counter ion linked via a C_4_H_6_ moiety (Scheme 1, top row). To elucidate the role of the bound anion in the nanostructuring of these ZILs, we also prepared a set of regular ILs with a [CH_3_SO_3_]^−^ anion and a corresponding imidazolium-based cation (Scheme 1, middle row). Similar to the previous studies [39,40,41,43,44,45,56], stable nitroxide radicals N1 and TEMPO-D_18_ were used as spin probes in EPR spectroscopy (Scheme 1, bottom row).

**Figure 1:**
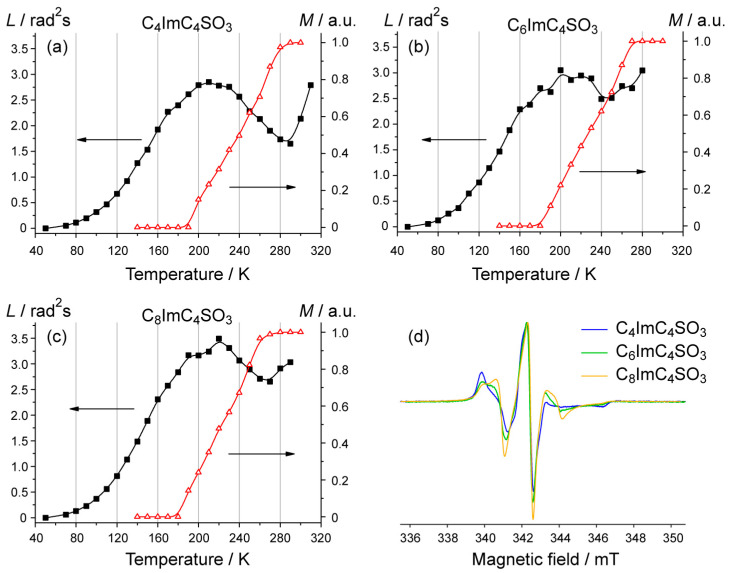
(**a**–**c**) The temperature dependence of the motional parameter, *L* ≈ 10^11^ < α^2^ > τ_c_, for nitroxide radical N1 (filled symbols, left axes) and the mobile fraction *M* for nitroxide radical TEMPO-D_18_ (open symbols, right axes) dissolved in selected zwitter ILs. (**d**) CW EPR spectra of nitroxide radical TEMPO-D_18_ in selected zwitter ILs at room temperature. Solid lines have been added as a guide.

For each studied ZIL and corresponding IL analogue, we performed continuous wave (CW) and pulse EPR studies and obtained temperature dependences of mobile fraction *M*(*T*) and parameters of molecular librations *L*(*T*) (see Experimental part, Methodology section for details). Figure 1a–c (filled symbols, left axes) show the *L*(*T*) dependencies obtained in the studied ZILs: C_4_ImC_4_SO_3_, C_6_ImC_4_SO_3_, and C_8_ImC_4_SO_3_. Notably, the common types of characteristic *L*(*T*) behaviors were observed. Our previous studies on imidazolium-based ILs [C_1_C_n_Im][BF_4_] (n = 0–12) have demonstrated that alkyl chain length has a major impact on the shape and characteristics of the *L*(*T*) curve [41]. Namely, gradual changes of *L*(*T*) vs. n have been observed for n = 3–10, including the effect of odd/even number of C-atoms in the chain. For n > 10, a mesophase instead of a glassy state occurs, leading to the suppression of the anomaly. Altogether, it was clearly demonstrated that alkyl chains do govern the presence and character of structural anomaly [41].

As was described in our previous work [39,40,41,42,43,44,45,46], the consideration of data obtained by two EPR methods (pulse and CW EPR) allows fruitful insights on heterogeneous structure of ILs to be gained. Stochastic molecular librations reflect a pronounced mobility of the probes located in the glassy matrix, whereas librations do not occur in a crystalline state. At the same time, CW EPR spectra of nitroxide probes are observable at any state of solvent: this refers to a liquid state, where the diffusive rotation of radicals occurs, and also applies to a crystalline state, where radicals are immobilized. According to the procedure described in detail in our previous studies [39,40,41,42,43,44,45,46], we obtained the CW EPR spectra of the dissolved probe, TEMPO-D_18_, and simulated each spectrum as a superposition of the immobile and mobile fractions. Figure 1a–c (empty symbols, right axes) show the temperature dependence of the mobile fraction, *M*(*T*), for selected ZILs.

In all cases, ZILs demonstrate a structural anomaly, and the formation of the mobile fraction, *M*(*T*), begins at temperatures close to a local maximum of *L*(*T*). This implies the coexistence of two microenvironments in the anomaly region: mobile probes yielding diffusive rotation—displayed in *M*(*T*), and the immobilized fraction that grows with temperature (displayed in *L*(*T*)). Since the motion regime of the spin probe is controlled by a solvent state, the two fractions of probes correspond to the two types of IL phases—semi-liquid and rigid ones—which is a direct manifestation of the heterogeneous microstructure of the solvent. Selected ZILs have very high viscosity at ambient temperature, which is clearly seen from the shape of CW EPR spectrum in Figure 1d, and the obtained rotational correlation times 5.6 ns, 3.4 ns, 3.0 ns at room temperature correspond to the local viscosity (estimated from the Einstein–Stokes–Debye equation [64]) of 141 cP, 94 cP, and 77 cP for n = 4, 6, 8 in C_n_C_4_ImSO_3_, respectively. Such values are an order of magnitude larger compared to imidazolium ILs at the same temperature, as was studied in our previous work [65].

**Figure 2:**
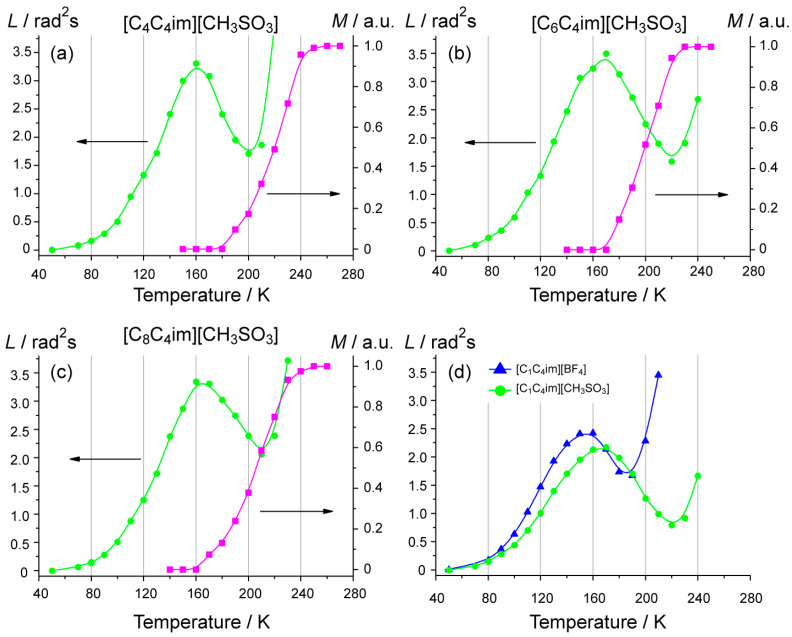
(**a**–**c**) The temperature dependence of the motional parameter, *L* ≈ 10^11^ < α^2^ > τ_c_, for nitroxide radical N1 (filled symbols, left axes) and the mobile fraction *M* for nitroxide radical TEMPO-D_18_ (open symbols, right axes) dissolved in selected [C_n_C_4_mim][CH_3_SO_3_] ILs. (**d**) *L*(*T*) curves for [C_1_C_4_mim][CH_3_SO_3_] and [C_1_C_4_im]BF_4_. Solid lines have been added as a guide.

To investigate the influence of the bound anion on the structural anomaly in ZILs, we synthesized the closest structural analogues—corresponding ILs with separate SO_3_^−^ anions (Scheme 1, Experimental). Since these ILs have not been investigated previously using EPR, we collected *L*(*T*) and *M*(*T*) curves for this series [C_n_C_4_mim][CH_3_SO_3_] first (Figure 2). One can notice that the general trends of *L*(*T*) and *M*(*T*) are similar to those previously obtained for other ILs, and both functions behave in the expected manner (Figure 2a–c). However, in the case of [CH_3_SO_3_] with symmetric cation, there is a mismatch between the local maximum of *L*(*T*) and onset of *M*(*T*). Moreover, one observes an irregular behavior in the vicinity of the *L*(*T*) maximum for [C_6_C_4_mim][CH_3_SO_3_]—locally reproducible curvature around 160 K—which is very similar to the previously observed odd–even effect in the [C_n_mim]BF_4_ series, where it took place for odd n [41]. Furthermore, it is interesting to clarify the influence of the relatively large [CH_3_SO_3_]^−^ anion on the characteristics of the anomaly. Figure 2d compares *L*(*T*) curves for [C_1_C_4_mim][CH_3_SO_3_] and [C_1_C_4_im][BF_4_]. It appears that the voluminous [CH_3_SO_3_]^−^ anion effectively increases the amplitude of the anomaly (Δ*L* between local maximum and minimum of *L*(*T*)), thus making it more prominent. A similar anomaly increase was previously observed for C2-methylated ILs [40,66].

**Figure 3:**
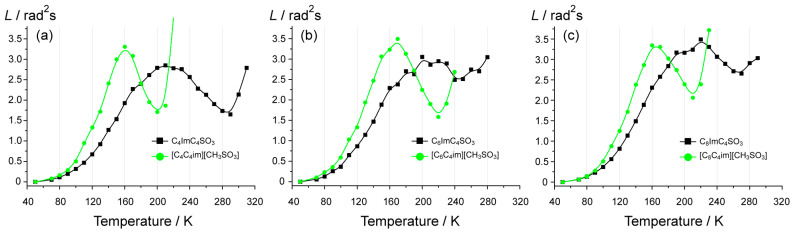
(**a**–**c**) The temperature dependence of the motional parameter, *L* ≈ 10^11^ < α^2^ > τ_c_, for nitroxide radical N1 dissolved in selected [C_n_C_4_mim][CH_3_SO_3_] and C_n_ImC_4_SO_3_ ILs. Solid lines have been added as a guide.

Finally we compared *L*(*T*) of ZILs C_n_ImC_4_SO_3_ with corresponding ILs [C_n_C_4_im][CH_3_SO_3_] (Figure 3a–c). Evidently, the anomaly curve in ZILs is more prolonged and smoothed. The local maximum of *L*(*T*) in each ZIL is spread over ~ 40 K compared to ~20 K in the non-zwitter analogue. We assume that the attachment of the [CH_3_SO_3_]^−^ anion to the alkyl chain significantly suppresses the alkyl chain dynamics: the local rearrangements of alkyl chains require more thermal energy to become activated. Qualitatively, the massive [CH_3_SO_3_]^−^ anion restricts rearrangements, acting like “a ball on a chain”. This also correlates with higher *T*_g_ values in ZILs compared to the corresponding ILs and might be the main cause of such a correlation (DSC data are presented in Supplementary Materials). Based on the *L*(*T*) data, the *T*_g_ points roughly coincide with the local minima of *L*(*T*) curves, which mark the transition from small-angle librations (*T* < *T*_g_) to a large-scale diffusive rotation (*T* > *T*_g_), in good agreement with our previous research [39,41,43,44,45].

**Figure 4:**
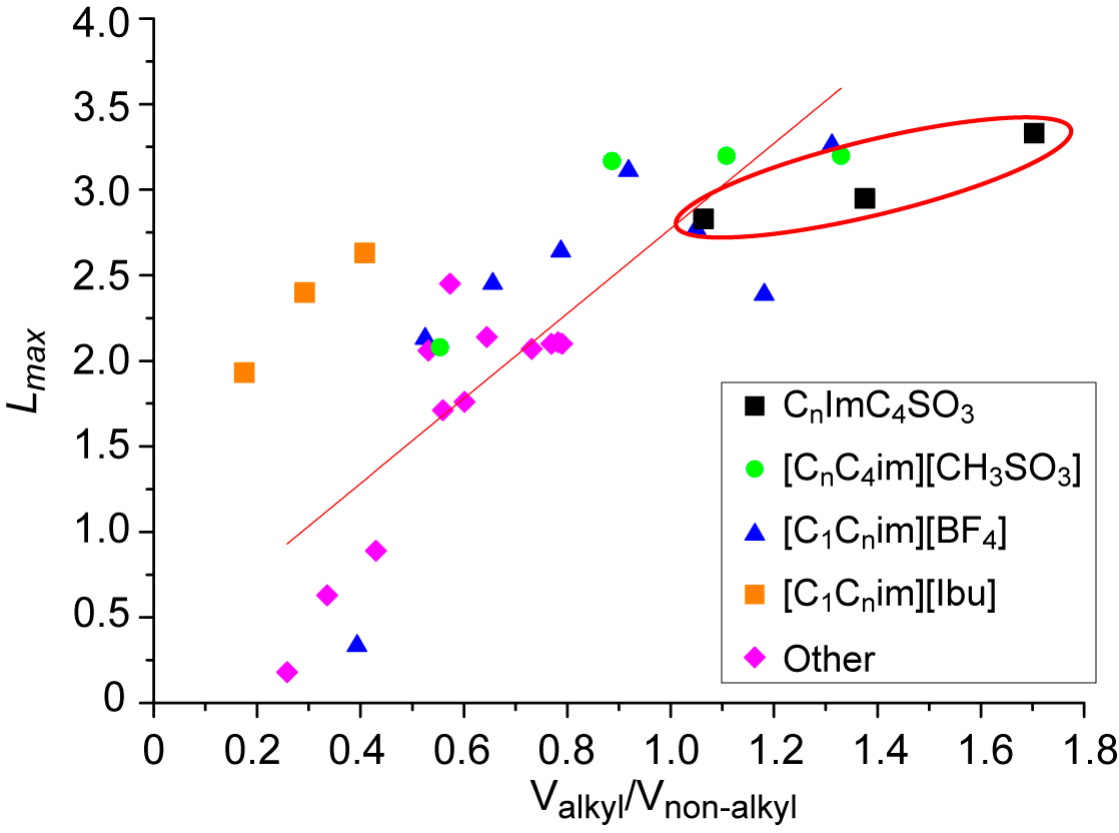
The maximum values of librations, *L*_max_, vs. the ratio of alkyl-chain volume to the volume of rest of the IL–ion couple. The red line shows the linear fit of the presented data, excluding results for [Ibu]^−^ and C_n_ImC_4_SO_3_.

It is worthwhile to correlate the peculiarities of *L*(*T*) observed in ZILs here and all *L*(*T*) trends found by us previously for common non-zwitter ILs. In our previous studies on common ILs, we have demonstrated that the maximum value of the libration parameter *L*_max_ correlates with the rigidity of the glassy matrix [39,40,41,42,43,44,45,46]. Thus, there is a correlation between the relative volume of the alkyl chain and the *L*_max_ value. Figure 4 shows the data collected in this and previous studies of structural anomalies in glassy ILs [39,40,41,42,43,44,45,46]. The volumes of the [BF_4_]^−^, [PF_6_]^−^, [Cl]^−^, [Br]^−^, [CF_3_SO_3_]^−^, and [C_n_mim]^+^ ions were taken from the literature [61]. The volumes of the anions in selected species were estimated by the “atoms in molecules” (AIM) partitioning of electron density distributions [61]. The geometries of the selected ions and zwitterions were optimized using DFT-B3LYP/def2-TZVP calculations in the Orca software [62] (see Supplementary Materials for more information). Subsequently, the density distribution was obtained using AIM partitioning. Using the Multiwfn program [63], Bader analysis was performed and Van der Waals volumes of selected species were calculated. The red line corresponds to the main trend for the collected data: since the radical is embedded in alkyl-rich nanodomains [41,44,45], the larger the volume of alkyl chains, the softer the glassy matrix around the radical, allowing for more pronounced librations. It is clear that the data points for [C_n_C_4_Im][CH_3_SO_3_]^−^ ILs belong to the main trend, whereas the points for ZILs C_n_ImC_4_SO_3_ are clear outliers, implying a different underlying nature of heterostructures formed around the spin probe. A similar situation has previously been observed for ILs with [Ibu]^−^ ions [45]. We assume that [C_1_C_n_Im][Ibu] ILs form complex anion/cation micelle-like environments, because such ILs include alkyl chains not only in cations, but also in anions. The difference of C_n_ImC_4_SO_3_ ZILs from IL analogues is of another kind: ZILs have cations and anions combined in a single molecule. However, in both cases, the structure imposes specifics on self-organization into nanostructures; therefore, the nanostructures formed are different from those in common (e.g., imidazolium-based) ILs. Note that in the case of C_n_ImC_4_SO_3_ the increase in the alkyl volume of ~70% (V_alkyl_/V_non-alkyl_ ≈ 1 ÷ 1.7) does not lead to significant rise in *L*_max_, in contrast to the main trend. Possibly, the molecules of C_n_ImC_4_SO_3_ tend to form relatively dense liquid-crystal-like environments; therefore, the increase in non-polar volume has a weaker impact on the micelle-like apolar domains, where the spin probe tends to reside.

Overall, we suppose that combining cations and anions in a single molecule, which takes place for ZILs, leads to certain restrictions in mutual diffusion of cations/anions, and thus to some modification in segregation of polar and apolar moieties and the structure of the heterogeneities formed. The segregation of polar and apolar moieties takes place in common ILs with “separate” cations and anions and has been broadly discussed in the literature previously [67,68,69]. Since the underlying interactions are also valid for ZILs, one might expect similar trends for segregation in ZILs. However, sterical restrictions for mutual diffusion of cations and anions, which are imposed by binding them into a single molecule, are expected to modify local nanostructures and influence their character monitored via *L*(*T*) and *M*(*T*) in our approach. Note that we recently observed somewhat similar sterical influence when a radical probe was attached to a (non-zwitterionic) IL: it appeared that the location (distribution of locations) of the free radical probe and location of the attached probe were slightly different, as was manifested in smoothed *L*(*T*) curves with prolonged plateaus [46]. In that case, the migration of the radical probe was restricted, whereas, in the current situation with ZILs, the mutual arrangement of zwitterions is restricted, leading to a qualitatively similar manifestations.

## 4. Conclusions and Outlook

In this work, we continued our investigation of fundamentally interesting and potentially promising phenomena—structural anomalies in ionic liquid glasses, which occur near the corresponding glass-transition temperatures. For the first time, we investigated such anomalies in zwitterionic ILs, where the cation and anion are combined in one molecule, thus restricting to some extent the segregation of polar/apolar moieties well known for common ILs [67,68,69]. First, we observed structural anomalies for ZIL glasses, which are generally similar to those in common ILs. In all studied cases, the amplitude of anomaly in ZIL was comparable to analogous non-zwitterionic IL, and in all studied cases, the temperature range of the anomaly was broader in ZILs. This was qualitatively rationalized by the above-mentioned sterical restrictions, resulting also in a broader distribution of local environments. In addition, glass transition temperatures in all studied ZILs were noticeably higher than those in corresponding IL analogues. Altogether, such changes in the anomaly properties are advantageous, since the temperature range moves closer to the room temperature, and the anomaly itself becomes more pronounced.

We proposed previously that such structural anomalies can potentially be used in the design of smart nanomaterials when ILs are incorporated into the porous matrices [70,71,72,73]. Another potential application refers to the cryopreservation of biomolecules [74,75]. In both cases, shifting the anomaly toward room temperature appears advantageous. Although more work is required to achieve designer guidelines, the present work introduces an appealing approach for tuning structural anomalies by combining cations and anions in zwitterions, which is an important step toward potential applications.

## Data Availability

Not applicable.

## Supplementary Materials

The following supporting information can be downloaded at: https://www.mdpi.com/article/10.3390/nano13152164/s1. Refs. [76,77] are cited in Supplementary Materials.

## References

[1] Welton T. (1999). Room-Temperature Ionic Liquids. Solvents for Synthesis and Catalysis. Chem. Rev..

[2] Hallett J.P., Welton T. (2011). Room-Temperature Ionic Liquids: Solvents for Synthesis and Catalysis. 2. Chem. Rev..

[3] Krossing I., Slattery J.M., Daguenet C., Dyson P.J., Oleinikova A., Weingärtner H. (2006). Why are ionic liquids liquid? A simple explanation based on lattice and solvation energies. J. Am. Chem. Soc..

[4] Walter K. (1948). The Nature of the Glassy State and the Behaviour of Liquids at Low Temperatures–Walter Kauzmann. Chem. Rev..

[5] Pârvulescu V.I., Hardacre C. (2007). Catalysis in ionic liquids. Chem. Rev..

[6] Tarasova N.P., Smetannikov Y.V., Zanin A.A. (2010). Ionic liquids in the synthesis of nanoobjects. Russ. Chem. Rev..

[7] Greaves T.L., Drummond C.J. (2008). Protic ionic liquids: Properties and applications. Chem. Rev..

[8] Dai C., Zhang J., Huang C., Lei Z. (2017). Ionic Liquids in Selective Oxidation: Catalysts and Solvents. Chem. Rev..

[9] Dong K., Liu X., Dong H., Zhang X., Zhang S. (2017). Multiscale Studies on Ionic Liquids. Chem. Rev..

[10] Ferraz R., Branco L.C., Prudêncio C., Noronha J.P., Petrovski Ž. (2011). Ionic liquids as active pharmaceutical ingredients. ChemMedChem.

[11] Watanabe M., Thomas M.L., Zhang S., Ueno K., Yasuda T., Dokko K. (2017). Application of Ionic Liquids to Energy Storage and Conversion Materials and Devices. Chem. Rev..

[12] Wang Y., Voth G.A. (2005). Unique spatial heterogeneity in ionic liquids. J. Am. Chem. Soc..

[13] Hayes R., Warr G.G., Atkin R. (2015). Structure and Nanostructure in Ionic Liquids. Chem. Rev..

[14] Wang Y.-L.L., Li B., Sarman S., Mocci F., Lu Z.Y., Yuan J., Laaksonen A., Fayer M.D. (2020). Microstructural and Dynamical Heterogeneities in Ionic Liquids. Chem. Rev..

[15] Zhao D., Wu M., Kou Y., Min E. (2002). Ionic liquids: Applications in catalysis. Catal. Today.

[16] Weingärtner H. (2008). Understanding Ionic Liquids at the Molecular Level: Facts, Problems, and Controversies. Angew. Chem. Int. Ed..

[17] Moshikur R., Goto M. (2021). Application of Ionic Liquids in Drug Delivery.

[18] Ford L., Tay E., Nguyen T.H., Williams H.D., Benameur H., Scammells P.J., Porter C.J.H. (2020). API ionic liquids: Probing the effect of counterion structure on physical form and lipid solubility. RSC Adv..

[19] Claus J., Sommer F.O., Kragl U. (2018). Ionic liquids in biotechnology and beyond. Solid State Ion..

[20] Ohno H., Yoshizawa-Fujita M., Kohno Y. (2018). Design and properties of functional zwitterions derived from ionic liquids. Phys. Chem. Chem. Phys..

[21] Bühler G., Zharkouskaya A., Feldmann C. (2008). Ionic liquid based approach to nanoscale functional materials. Solid State Sci..

[22] Kang X., Sun X., Han B. (2016). Synthesis of Functional Nanomaterials in Ionic Liquids. Adv. Mater..

[23] Ranjan P., Yadav S., Sadique M.A., Khan R. (2021). Nanomaterials for the Electrochemical Biosensors. Biosensors.

[24] Yoshizawa M., Ohno H. (2004). Anhydrous proton transport system based on zwitterionic liquid and HTFSI. Chem. Commun..

[25] Suzanowicz A.M., Mei C.W., Mandal B.K. (2022). Approaches to Combat the Polysulfide Shuttle Phenomenon in Li–S Battery Technology. Batteries.

[26] Di Donato G., Ates T., Adenusi H., Varzi A., Navarra M.A., Passerini S. (2022). Electrolyte Measures to Prevent Polysulfide Shuttle in Lithium-Sulfur Batteries. Batter. Supercaps.

[27] Ren W., Ma W., Zhang S., Tang B. (2019). Recent advances in shuttle effect inhibition for lithium sulfur batteries. Energy Storage Mater..

[28] Zhang Z., Zhang P., Liu Z., Du B., Peng Z. (2020). A Novel Zwitterionic Ionic Liquid-Based Electrolyte for More Efficient and Safer Lithium-Sulfur Batteries. ACS Appl. Mater. Interfaces.

[29] Yoshizawa M., Hirao M., Ito-Akita K., Ohno H. (2001). Ion conduction in zwitterionic-type molten salts and their polymers. J. Mater. Chem..

[30] Wu B., Kuroda K., Takahashi K., Castner E.W. (2018). Structural analysis of zwitterionic liquids vs. homologous ionic liquids. J. Chem. Phys..

[31] Biswas Y., Ghosh P., Mandal T.K. (2018). Chemical Tuning of Zwitterionic Ionic Liquids for Variable Thermophysical Behaviours, Nanostructured Aggregates and Dual-Stimuli Responsiveness. Chem.—A Eur. J..

[32] Hermanutz F., Vocht M.P., Panzier N., Buchmeiser M.R. (2019). Processing of Cellulose Using Ionic Liquids. Macromol. Mater. Eng..

[33] Wang H., Gurau G., Rogers R.D. (2012). Ionic liquid processing of cellulose. Chem. Soc. Rev..

[34] Brandt A., Gräsvik J., Hallett J.P., Welton T. (2013). Deconstruction of lignocellulosic biomass with ionic liquids. Green Chem..

[35] Sharma G., Takahashi K., Kuroda K. (2021). Polar zwitterion/saccharide-based deep eutectic solvents for cellulose processing. Carbohydr. Polym..

[36] Kuroda K., Satria H., Miyamura K., Tsuge Y., Ninomiya K., Takahashi K. (2017). Design of Wall-Destructive but Membrane-Compatible Solvents. J. Am. Chem. Soc..

[37] Satria H., Kuroda K., Endo T., Takada K., Ninomiya K., Takahashi K. (2017). Efficient Hydrolysis of Polysaccharides in Bagasse by in Situ Synthesis of an Acidic Ionic Liquid after Pretreatment. ACS Sustain. Chem. Eng..

[38] Huet G., Araya-Farias M., Alayoubi R., Laclef S., Bouvier B., Gosselin I., Cézard C., Roulard R., Courty M., Hadad C. (2020). New biobased-zwitterionic ionic liquids: Efficiency and biocompatibility for the development of sustainable biorefinery processes. Green Chem..

[39] Ivanov M.Y., Fedin M.V. (2018). Nanoscale heterogeneities in ionic liquids: Insights from EPR of spin probes. Mendeleev Commun..

[40] Ivanov M.Y., Prikhod’ko S.A., Adonin N.Y., Kirilyuk I.A., Adichtchev S.V., Surovtsev N.V., Dzuba S.A., Fedin M.V. (2018). Structural Anomalies in Ionic Liquids near the Glass Transition Revealed by Pulse EPR. J. Phys. Chem. Lett..

[41] Bakulina O.D., Ivanov M.Y., Prikhod’ko S.A., Pylaeva S., Zaytseva I.V., Surovtsev N.V., Adonin N.Y., Fedin M.V. (2020). Nanocage formation and structural anomalies in imidazolium ionic liquid glasses governed by alkyl chains of cations. Nanoscale.

[42] Ivanov M.Y., Poryvaev A.S., Polyukhov D.M., Prikhod’ko S.A., Adonin N.Y., Fedin M.V. (2020). Nanoconfinement effects on structural anomalies in imidazolium ionic liquids. Nanoscale.

[43] Ivanov M.Y., Prikhod’ko S.A., Bakulina O.D., Kiryutin A.S., Adonin N.Y., Fedin M.V. (2021). Validation of structural grounds for anomalous molecular mobility in ionic liquid glasses. Molecules.

[44] Ivanov M.Y., Bakulina O.D., Alimov D.V., Prikhod’ko S.A., Veber S.L., Pylaeva S., Adonin N.Y., Fedin M.V. (2021). Inherent heterogeneities and nanostructural anomalies in organic glasses revealed by EPR. Nanoscale.

[45] Bakulina O.D., Ivanov M.Y., Alimov D.V., Prikhod’ko S.A., Adonin N.Y., Fedin M.V. (2022). Active Pharmaceutical Ingredient-Ionic Liquids (API-ILs): Nanostructure of the Glassy State Studied by Electron Paramagnetic Resonance Spectroscopy. Molecules.

[46] Ivanov M.Y., Bakulina O.D., Polienko Y.F., Kirilyuk I.A., Prikhod’ko S.A., Adonin N.Y., Fedin M.V. (2023). Radical ionic liquid: An efficient self-probe to study heterogeneous structure in glassy state using EPR spectroscopy. J. Mol. Liq..

[47] Ivanov M.Y., Prikhod’ko S.A., Adonin N.Y., Fedin M.V. (2019). Structural Anomalies in Binary Mixtures of Ionic Liquid [Bmim]BF 4 with Water Studied by EPR. J. Phys. Chem. B.

[48] Stoll S., Schweiger A. (2006). EasySpin, a comprehensive software package for spectral simulation and analysis in EPR. J. Magn. Reson..

[49] Kattnig D.R., Akdogan Y., Lieberwirth I., Hinderberger D. (2013). Spin probing of supramolecular structures in 1-butyl-3-methyl-imidazolium tetrafluoroborate/water mixtures. Mol. Phys..

[50] Akdogan Y., Heller J., Zimmermann H., Hinderberger D. (2010). The solvation of nitroxide radicals in ionic liquids studied by high-field EPR spectroscopy. Phys. Chem. Chem. Phys..

[51] Kattnig D.R., Akdogan Y., Bauer C., Hinderberger D. (2012). High-field EPR spectroscopic characterization of spin probes in aqueous ionic liquid mixtures. Z. Phys. Chem..

[52] Mladenova B.Y., Chumakova N.A., Pergushov V.I., Kokorin A.I., Grampp G., Kattnig D.R. (2012). Rotational and translational diffusion of spin probes in room-temperature ionic liquids. J. Phys. Chem. B.

[53] Mladenova B.Y., Kattnig D.R., Grampp G. (2011). Room-temperature ionic liquids discerned via nitroxyl spin probe dynamics. J. Phys. Chem. B.

[54] Strehmel V. (2012). Radicals in Ionic Liquids. ChemPhysChem.

[55] Stoesser R., Herrmann W., Zehl A., Strehmel V., Laschewsky A. (2006). ESR spin probes in ionic liquids. ChemPhysChem.

[56] Bakulina O.D., Ivanov M.Y., Prikhod’ko S.A., Adonin N.Y., Fedin M.V. (2021). EPR study of nanostructuring in protic ionic liquids [PriNH_3_]NO_3_ and [BuNH3]NO_3_. Russ. Chem. Bull. Int. Ed..

[57] Erilov D.A., Bartucci R., Guzzi R., Marsh D., Dzuba S.A., Sportelli L. (2004). Librational motion of spin-labeled lipids in high-cholesterol containing membranes from echo-detected EPR spectra. Biophys. J..

[58] Isaev N.P., Dzuba S.A. (2008). Fast Stochastic Librations and Slow Rotations of Spin Labeled Stearic Acids in a Model Phospholipid Bilayer at Cryogenic Temperatures. J. Phys. Chem. B.

[59] Syryamina V.N., Dzuba S.A. (2017). Dynamical Transitions at Low Temperatures in the Nearest Hydration Shell of Phospholipid Bilayers. J. Phys. Chem. B.

[60] Dzuba S.A. (2000). Libration motion of guest spin probe molecules in organic glasses: CW EPR and electron spin echo study. Spectrochim. Acta—Part A Mol. Biomol. Spectrosc..

[61] Nelyubina Y.V., Shaplov A.S., Lozinskaya E.I., Buzin M.I., Vygodskii Y.S. (2016). A New Volume-Based Approach for Predicting Thermophysical Behavior of Ionic Liquids and Ionic Liquid Crystals. J. Am. Chem. Soc..

[62] Neese F. (2012). The ORCA program system. Wiley Interdiscip. Rev. Comput. Mol. Sci..

[63] Lu T., Chen F. (2012). Multiwfn: A multifunctional wavefunction analyzer. J. Comput. Chem..

[64] Andreozzi L., Di Schino A., Giordano M., Leporini D. (1997). Evidence of a fractional Debye-Stokes-Einstein law in supercooled o-terphenyl. Europhys. Lett..

[65] Ivanov M.Y., Veber S.L., Prikhod’ko S.A., Adonin N.Y., Bagryanskaya E.G., Fedin M.V., Prikhod’ko S.A., Adonin N.Y., Bagryanskaya E.G., Fedin M.V. (2015). Probing Microenvironment in Ionic Liquids by Time-Resolved EPR of Photoexcited Triplets. J. Phys. Chem. B.

[66] Ivanov M.Y., Prikhod’Ko S.A., Adonin N.Y., Bagryanskaya E.G., Fedin M.V. (2017). Influence of C2-Methylation of Imidazolium Based Ionic Liquids on Photoinduced Spin Dynamics of the Dissolved ZnTPP Studied by Time-Resolved EPR. Z. Phys. Chem..

[67] Rocha M.A.A., Neves C.M.S.S., Freire M.G., Russina O., Triolo A., Coutinho J.A.P., Santos L.M.N.B.F. (2013). Alkylimidazolium based ionic liquids: Impact of cation symmetry on their nanoscale structural organization. J. Phys. Chem. B.

[68] Russina O., Triolo A., Gontrani L., Caminiti R., Xiao D., Hines L.G., Bartsch R.A., Quitevis E.L., Plechkova N., Seddon K.R. (2009). Morphology and intermolecular dynamics of 1-alkyl-3-methylimidazolium bis{(trifluoromethane)sulfonyl}amide ionic liquids: Structural and dynamic evidence of nanoscale segregation. J. Phys. Condens. Matter.

[69] Triolo A., Russina O., Bleif H.J., Di Cola E. (2007). Nanoscale segregation in room temperature ionic liquids. J. Phys. Chem. B.

[70] Avila J., Clark R., Pádua A.A.H., Costa Gomes M. (2022). Porous ionic liquids: Beyond the bounds of free volume in a fluid phase. Mater. Adv..

[71] Durak O., Zeeshan M., Habib N., Gulbalkan H.C., Alsuhile A.A.A.M., Caglayan H.P., Kurtoğlu-Öztulum S.F., Zhao Y., Haslak Z.P., Uzun A. (2022). Composites of porous materials with ionic liquids: Synthesis, characterization, applications, and beyond. Microporous Mesoporous Mater..

[72] Barrulas R.V., Zanatta M., Casimiro T., Corvo M.C. (2021). Advanced porous materials from poly(ionic liquid)s: Challenges, applications and opportunities. Chem. Eng. J..

[73] Avila J., Červinka C., Dugas P.Y., Pádua A.A.H., Costa Gomes M. (2021). Porous Ionic Liquids: Structure, Stability, and Gas Absorption Mechanisms. Adv. Mater. Interfaces.

[74] Takekiyo T., Ishikawa Y., Yoshimura Y. (2017). Cryopreservation of proteins using ionic liquids: A case study of cytochrome c. J. Phys. Chem. B.

[75] Yoshimura Y., Takekiyo T., Mori T. (2016). Structural study of lysozyme in two ionic liquids at cryogenic temperature. Chem. Phys. Lett..

[76] Cai J., Liu J., Mu S., Liu J., Hong J., Zhou X., Ma Q., Shi L. (2020). Corrosion inhibition effect of three imidazolium ionic liquids on carbon steel in chloride contaminated environment. Int. J. Electrochem. Sci..

[77] Dupont J., Consorti C.S., Suarez P.A.Z., De Souza R.F. (2002). Preparation of 1-butyl-3-methyl imidszolium-based room temperature ionic liquids. Org. Synth..

