# Supplementary material for: Effects of Zwitterions on Structural Anomalies in Ionic Liquid Glasses Studied by EPR
Source: Nanomaterials (Basel). 2023 Jul 26;13(15):2164. doi: 10.3390/nano13152164 (PMC10420841; doi:10.3390/nano13152164)
Supplement: Supplementary file 1 [file nanomaterials-13-02164-s001.zip › nanomaterials-2522416-supplementary.pdf]

SUPPORTING INFORMATION

Effects of zwitterions on structural anomalies in ionic liquids studied with EPR

Olga D. Bakulina[1,2], *Mikhail Yu. Ivanov*[1,2*], *Sergey A. Prikhod'ko*[3], *Nicolay Yu. Adonin*[3] *and Matvey V. Fedin*[1,2*]

[1] International Tomography Center SB RAS, Institutskaya Street 3a, 630090 Novosibirsk, Russia

[2] Novosibirsk State University, Pirogova Street 2, 630090 Novosibirsk, Russia

[3] Boreskov Institute of Catalysis SB RAS, Lavrentiev Avenue 5, 630090 Novosibirsk, Russia

* Correspondence: michael.ivanov@tomo.nsc.ru (M.Y.I.); mfedin@tomo.nsc.ru (M.V.F.)

Table of Contents

Chemicals....................S2

Synthesis of ionic liquids....................S2

The NMR spectral data of ionic liquids....................S3

DSC measurements....................S4

Quantum chemical calculations....................S5

EPR of stochastic molecular librations....................S12

Continuous wave EPR....................S14

References....................S20

Chemicals

Alkyl bromides, N-methyl imidazole, 1-butyl-3-methyl imidazolium chloride, 1,4-butane sultone, and all standard chemicals were obtained from commercial sources. Methanesulfonic acid sodium salt was prepared by neutralizing a solution of methanesulfonic acid with an equimolar amount of sodium hydroxide solution, followed by drying in high vacuum.

Synthesis of ionic liquids

N-Alkyl imidazoles (general procedure)

N-alkylimidazoles $C_nH_{2n+1}Im$ (n = 4, 6, 8) were obtained by using the analogy with the literature method [S1] from imidazole and corresponding alkyl bromides, and purified by distillation in vacuum. 1-butyl-3-methyl imidazolium chloride and 1-butyl-3-alkyl imidazolium chlorides (alkyl = butyl, hexyl or octyl) were obtained by using the analogy with the literature method [S2] from N-methylimidazole or N-butylimidazole and corresponding alkyl chloride.

1-Alkylimidazolium-3-butansulfonates (general procedure)

A 100 ml round-bottomed flask fitted with magnetic stirrer bar and reflux condenser was charged with 59 mmol $C_nH_{2n+1}Im$, 8.45 g (62 mmol) of 1,4-butansultone and 40 ml of acetonitrile. The mixture was refluxed for 12 hours. After that, about 80% of the solvent was distilled off and 50 ml of diethyl ether was added. The product was filtered, washed with diethyl ether and dried in high vacuum (10^{-3} bar) overnight. Yield of products was 88-95 %.

1-Butyl-3-alkyl imidazolium methanesulphonates (general procedure)

A 100 ml round-bottomed flask fitted with a magnetic stirrer bar and reflux condenser was charged with 36.5 mmol of 1-butyl-3-alkyl imidazolium chloride and 20 ml of acetonitrile. After complete dissolution, 7.36 g (54.8 mmol) of solution of CH_3SO_3K in 5 ml of water was added. The mixture was stirred for 2 hours. During this time, the mixture was divided into 2 layers. The water–salt layer (lower) was separated and extracted with 10 ml of acetonitrile. The extract was combined with organic phase and the second portion of CH_3SO_3K (7.36 g, 54.8 mmol) in 5 ml of water was added to the filtrate and the resulting mixture was stirred for 3 hours. The mixture was divided into 2 layers. The water–salt layer (lower) was separated and extracted with 10 ml of acetonitrile. The extract was combined with organic phase, dried with $MgSO_4$, and evaporated. The product was dissolved in 20 ml of dichloromethane to separate impurities from the starting salt. The solid was filtered and washed with 10 ml of dichloromethane. The filtrate was evaporated and product was dried in high vacuum (10^{-3} bar) at 80 °C for 6 hours. Yield of products was 70-85 %.

1-Butyl-3-methyl imidazolium methanesulphonate

A 100 ml round-bottomed flask fitted with magnetic stirrer bar and reflux condenser was charged by 5.0 g (28.6 mmol) of 1-butyl-3-methyl imidazolium chloride, 4.96 g (42.9 mmol) of CH_3SO_3Na and 50 ml of acetonitrile. The mixture was stirred for 6 hours at 60 °C. The solid was filtered and washed with 10 ml of acetonitrile. The second portion of CH_3SO_3Na (4.96 g, 42.9 mmol) was added to the filtrate and the resulting mixture was stirred for 3 hours at 60 °C. The solid was filtered and washed with 10 ml of acetonitrile. The filtrate was evaporated in vacuum. The residue was solved in 30 ml of dry dichloromethane (to remove excess of starting salt) and filtered. The filtrate was evaporated and the product was dried in high vacuum (10^{-3} bar) at 80 °C for 6 hours. Yield of product was 72 %.

The NMR spectral data of ionic liquids

The NMR spectra were recorded on a Bruker AVANCE 300 spectrometer (300.13 MHz) using CD_3CN as solvent (unless otherwise indicated). The chemical shifts are with reference to TMS.

1-Butyl-3-(4-sulfobutyl) imidazolium

^{1}H NMR: δ 8.99 (s, 1H, *H*-2); 7.47 (s, 1H, *H*-4); 7.37 (s, 1H, *H*-5); 4.23 (t, 2H, $^3J_{HH}$ 7.4 Hz, N-CH_2) 4.12 (t, 2H, $^3J_{HH}$ 7.4 Hz, N-CH_2); 2.61 (m, 2H, CH_2); 2.02 (m, 2H, CH_2); 1.65-1.95 (m, 4H, 2 CH_2); 1.29 (qt, 2H, $^3J_{HH}$ 7.4 Hz, $^3J_{HH}$ 7.5 Hz, CH_2); 0.92 (t, 3H, $^3J_{HH}$ 7.4 Hz, CH_3).

1-Hexyl-3-(4-sulfobutyl) imidazolium

^{1}H NMR: δ 9.18 (s, 1H, *H*-2); 7.77 (s, 2H, *H*-4, *H*-5); 4.17 (m, 4H, 2 N-CH_2); 2.49 (m, 4H, 2 CH_2); 2.49 (m, 4H, 2 CH_2); 1.68-1.99 (m, 4H, 2CH_2); 1.08-1.36 (m, 6H, ,3 CH_2); 0.84 (t, 3H, $^3J_{HH}$ 7.4 Hz, CH_3).

1-Octyl-3-(4-sulfobutyl) imidazolium

^{1}H NMR: δ 8.79 (s, 1H, *H*-2); 7.48 (s, 1H, *H*-4); 7.47 (s, 1H, *H*-5); 4.22 (t, 2H, $^3J_{HH}$ 7.5 Hz, N-CH_2) 4.16 (t, 2H, $^3J_{HH}$ 7.4 Hz, N-CH_2); 2.91 (m, 2H, CH_2); 2.00 (m, 2H, CH_2); 1.84 (m, 2H, CH_2); 1.71 (m, 2H, CH_2); 2.49 (m, 4H, 2 CH_2); 1.20-1.28 (m, 10H, ,5 CH_2); 0.82 (t, 3H, $^3J_{HH}$ 7.5 Hz, CH_3).

1-Butyl-3-methyl imidazolium methanesulfonate

^{1}H NMR: δ 9.92 (s, 1H, *H*-2); 7.348 (s, 1H, *H*-4); 7.36 (s, 1H, *H*-5); 4.19 (t, 2H, $^3J_{HH}$ 7.4 Hz, N-CH_2); 3.97 (s, 3H, NCH_3); 2.68 (s, 3H, CH_3SO_3); 1.79 (tt, 2H, $^3J_{HH}$ 7.5 Hz, $^3J_{HH}$ 7.4 Hz, 2-CH_2); 1.28 (qt, 2H, $^3J_{HH}$ 7.4 Hz, $^3J_{HH}$ 7.5 Hz, 3-CH_2); 0.86 (t, 3H, $^3J_{HH}$ 7.4 Hz, 4-CH_3).

1-Butyl-3-methyl imidazolium methanesulfonate

^{1}**H NMR**: δ 9.92 (s, 1H, *H*-2); 7.35 (s, 1H, *H*-4); 7.36 (s, 1H, *H*-5); 4.19 (t, 2H, $^{3}J_{HH}$ 7.4 Hz, N-CH_2); 3.97 (s, 3H, NCH_3); 2.68 (s, 3H, CH_3SO_3); 1.79 (tt, 2H, $^{3}J_{HH}$ 7.5 Hz, $^{3}J_{HH}$ 7.4 Hz, 2-CH_2); 1.28 (qt, 2H, $^{3}J_{HH}$ 7.4 Hz, $^{3}J_{HH}$ 7.5 Hz, 3-CH_2); 0.86 (t, 3H, $^{3}J_{HH}$ 7.4 Hz, 4-CH_3).

1,3-Dibutyl imidazolium methanesulfonate

^{1}**H NMR**: δ 9.68 (s, 1H, *H*-2); 6.57 (s, 1H, *H*-4); 6.65 (s, 1H, *H*-5); 3.94 (t, 4H, $^{3}J_{HH}$ 7.4 Hz, N-CH_2); 2.38 (s, 3H, CH_3SO_3); 1.51 (tt, 4H, $^{3}J_{HH}$ 7.5 Hz, $^{3}J_{HH}$ 7.4 Hz, 2-CH_2); 0.97 (qt, 4H, $^{3}J_{HH}$ 7.4 Hz, $^{3}J_{HH}$ 7.5 Hz, 3-CH_2); 0.57 (t, 6H, $^{3}J_{HH}$ 7.4 Hz, 4-CH_3).

1-Butyl-3-hexyl imidazolium methanesulfonate

^{1}**H NMR**: δ 9.63 (s, 1H, *H*-2); 7.35 (s, 1H, *H*-4); 7.31 (s, 1H, *H*-5); 3.93-3.99 (m, 4H, N-CH_2); 2.38 (s, 3H, CH_3SO_3); 1.49-1.57 (m, 4H, 2,2′-CH_2); 0.90-1.03 (m, 8H, 3′,3,4,5-CH_2); 0.57 (t, 3H, $^{3}J_{HH}$ 7.5 Hz, 4′-CH_3); 0.49 (t, 3H, $^{3}J_{HH}$ 7.2 Hz, 6-CH_3).

1-Butyl-3-octyl imidazolium methanesulfonate

^{1}**H NMR**: δ 9.18 (s, 1H, *H*-2); 7.14 (s, 1H, *H*-4); 7.10 (s, 1H, *H*-5); 3.63-3.69 (m, 4H, N-CH_2); 2.02 (s, 3H, CH_3SO_3); 1.14-1.30 (m, 4H, 2,2′-CH_2); 0.46-0.79 (m, 12H, 3′,3,4,5,6,7-CH_2); 0.25 (t, 3H, $^{3}J_{HH}$ 7.3 Hz, 4′-CH_3); 0.16 (t, 3H, $^{3}J_{HH}$ 7.1 Hz, 8-CH_3).

DSC measurements

DSC measurements were performed using DSC 204 F1 (NETZSCH) equipment at argon atmosphere with 30 ml/min flow rate. Samples were placed in the closed and pressed in an alumina melting pot with 25 μl volume. Setup calibration (on temperature and thermal capacity) was carried out using indium (99.999%) and zinc (99.8+%) purchased from Sigma Aldrich.

For sample 1, at the initial stage, heating was carried out to 120°C (heating rate 10°C/min), followed by rapid cooling (40°C/min) to a temperature of -60°C, then to -120°C at a rate of 20°C/min, and, after holding at the lowest possible temperature, the sample was heated at a rate of 1°C/min to -60°C and then to room temperature (25°C) at a rate of 10°C/min. Samples 2–4 were rapidly cooled from room temperature (40°C/min) to a temperature of -60°C, then to -120°C (20°C/min), held for 5 minutes at this temperature and heated to -60°C at a rate of 1°C/min and, further to room temperature (25°C) at a rate of 10°C/min. T_g values for samples 5-7 were obtained manually using the thermo-mechanical method. Results are presented at Table S1.

Table S1. Glass transition temperatures of studied samples determined by DSC.

№	Sample	State of aggregation	T_g onset, C	Inflection[1], C	ΔC_p , J/(g*K)
1	$[C_4C_1Im][CH_3SO_3]$	solid	**-69.5**	**-66.7**	**1.068**
2	$[C_4C_8Im][CH_3SO_3]$	Liquid	**-72.4**	**-69.4**	**1.225**
3	$[C_4C_6Im][CH_3SO_3]$	Liquid	**-81.3**	**-78.4**	**1.060**
4	$[C_4C_4Im][CH_3SO_3]$	Liquid	**-83.8**	**-81.4**	**1.567**
5	$C_4ImC_4SO_3$	Solid	**-8**	**-6**	
6	$C_6ImC_4SO_3$	Solid	**+4**	**+7**	
7	$C_8ImC_4SO_3$	Solid	–[2]	–[2]	

1 – The turning point between the beginning and the end of glass transition;
2 – At about 30°C, the substance passes into a crystalline form without the formation of a liquid phase.

Quantum chemical calculations

Quantum chemical calculations were performed using the Orca software [S3]. For geometry optimization of selected species, the DFT-B3LYP/def2-TZVP method was applied. The results of optimization are shown in Tables S2-S8.

Table S2. List of coordinates obtained during geometry optimization for $[C_4C_4Im]^+$.

Atom	x, Å	y, Å	y, Å
C	0.335968	-0.418900	-2.028656
N	1.073081	-1.528878	-1.968821
N	-0.612704	-0.472363	-1.092802
C	-1.645161	0.557103	-0.849950
C	-0.479290	-1.662871	-0.411463
C	0.573952	-2.323238	-0.959564
C	2.201944	-1.866111	-2.859954
H	-1.640000	0.770486	0.219821
H	-1.312707	1.455466	-1.367247

H	0.487848	0.395225	-2.716663
H	-1.127408	-1.934135	0.402866
H	1.009880	-3.274289	-0.710534
C	1.802830	-2.850228	-3.959456
H	1.003375	-2.407917	-4.562661
H	2.565858	-0.927082	-3.273328
H	1.389585	-3.750060	-3.496089
H	2.992191	-2.277451	-2.230307
C	2.981621	-3.238915	-4.861580
H	3.780394	-3.668618	-4.248552
H	2.641900	-4.044076	-5.516905
C	3.535328	-2.098676	-5.715995
H	3.979286	-1.301081	-5.115466
H	2.754256	-1.656536	-6.340001
H	4.317265	-2.464758	-6.382303
C	-3.029906	0.114666	-1.318861
H	-2.990856	-0.109379	-2.389176
H	-3.297160	-0.814578	-0.808101
C	-4.110651	1.169487	-1.044550
H	-4.130330	1.397699	0.025654
H	-5.077390	0.714280	-1.271455
C	-3.962968	2.458921	-1.852358
H	-4.804699	3.126007	-1.662550
H	-3.056252	3.013874	-1.598051
H	-3.942817	2.253110	-2.925705

Table S3. List of coordinates obtained during geometry optimization for $[C_6C_4Im]^+$.

Atom	x, Å	y, Å	y, Å
C	0.344345	-0.454521	-1.984429
N	1.053954	-1.583938	-1.971997
N	-0.628204	-0.538526	-1.075926
C	-1.642169	0.499838	-0.796277
C	-0.539478	-1.769229	-0.462200
C	0.511866	-2.422479	-1.022232
C	2.196277	-1.898222	-2.854260
H	-1.676354	0.630557	0.286305
H	-1.266817	1.425857	-1.228599
H	0.532085	0.393969	-2.619873
H	-1.214625	-2.070654	0.318969
H	0.919708	-3.396369	-0.817855
C	1.808564	-2.838209	-3.995261
H	1.026970	-2.365652	-4.598819
H	2.575215	-0.947943	-3.226320
H	1.375365	-3.747993	-3.571372
H	2.971677	-2.339836	-2.226548
C	3.001242	-3.211348	-4.885623
H	3.782809	-3.668970	-4.270436

H	2.666739	-3.991633	-5.572892
C	3.584601	-2.051443	-5.692510
H	4.024691	-1.277824	-5.058716
H	2.821460	-1.581190	-6.318269
H	4.376141	-2.405703	-6.353860
C	-3.018935	0.136259	-1.351344
H	-2.948833	0.022376	-2.437522
H	-3.316435	-0.836320	-0.949562
C	-4.091453	1.175979	-1.000517
H	-4.149536	1.283640	0.088285
H	-5.057084	0.769432	-1.313014
C	-3.900630	2.550698	-1.646241
H	-2.972409	3.014986	-1.292240
H	-3.792144	2.430121	-2.730792
C	-5.059856	3.509844	-1.361743
H	-5.991162	3.060205	-1.721155
H	-5.174277	3.624247	-0.278741
C	-4.875177	4.883151	-2.003392
H	-5.720111	5.537672	-1.782614
H	-3.971567	5.375757	-1.634262
H	-4.792324	4.804709	-3.090522

Table S4. List of coordinates obtained during geometry optimization for $[C_8C_4Im]^+$.

Atom	x, Å	y, Å	y, Å
C	0.351042	-0.504196	-1.952615
N	1.038397	-1.646809	-1.990871
N	-0.640564	-0.619333	-1.068533
C	-1.641971	0.419752	-0.749180
C	-0.587899	-1.884188	-0.524190
C	0.461677	-2.526167	-1.100202
C	2.193256	-1.934657	-2.865934
H	-1.705992	0.476745	0.338397
H	-1.235972	1.365089	-1.105439
H	0.567747	0.374617	-2.535228
H	-1.284696	-2.215160	0.225456
H	0.846685	-3.517996	-0.943203
C	1.817579	-2.824144	-4.050153
H	1.055621	-2.317993	-4.651491
H	2.585966	-0.973095	-3.192139
H	1.362957	-3.743165	-3.670832
H	2.954404	-2.408176	-2.243979
C	3.023916	-3.181471	-4.928550
H	3.783925	-3.676219	-4.314947
H	2.692580	-3.928090	-5.653658
C	3.644474	-2.000584	-5.675231
H	4.085258	-1.262144	-5.001237
H	2.903077	-1.490801	-6.296285

H	4.442993	-2.342472	-6.334685
C	-3.008134	0.123075	-1.365819
H	-2.909599	0.081397	-2.455012
H	-3.334645	-0.868394	-1.039333
C	-4.070942	1.158124	-0.974347
H	-4.133717	1.218403	0.117969
H	-5.039627	0.776686	-1.307996
C	-3.862322	2.557932	-1.558717
H	-2.915797	2.979769	-1.205481
H	-3.780937	2.483685	-2.649914
C	-5.004770	3.514704	-1.203290
H	-5.948305	3.060941	-1.524386
H	-5.068684	3.609308	-0.113243
C	-4.893815	4.910497	-1.826181
H	-5.823442	5.451110	-1.620712
H	-4.835788	4.818312	-2.917004
C	-3.719853	5.756360	-1.324538
H	-3.753886	5.804776	-0.230298
H	-2.770739	5.272756	-1.580348
C	-3.722876	7.174245	-1.894620
H	-2.875289	7.754310	-1.524125
H	-3.666046	7.162173	-2.986223
H	-4.635324	7.708469	-1.618043

Table S5. List of coordinates obtained during geometry optimization for $[CH_3SO_3]^-$.

Atom	x, Å	y, Å	y, Å
C	-2.134924	0.000267	-2.170990
H	-2.134924	1.029906	-1.817016
H	-3.026714	-0.515129	-1.817378
H	-1.243135	-0.515129	-1.817378
S	-2.134924	0.000335	-4.009189
O	-2.134924	1.418765	-4.376896
O	-0.906431	-0.709508	-4.376532
O	-3.363418	-0.709508	-4.376532

Table S6. List of coordinates obtained during geometry optimization for $C_4ImC_4SO_3$.

Atom	x, Å	y, Å	y, Å
C	0.457671	-0.774126	-1.783616
N	1.158314	-1.867727	-1.484883
N	-0.501725	-0.624520	-0.865869
C	-1.519034	0.434832	-0.890464
C	-0.410286	-1.658690	0.045358
C	0.633243	-2.436318	-0.345385
C	2.295314	-2.342988	-2.298644
H	-1.752637	0.685420	0.145689

H	-1.052612	1.306458	-1.345698
H	0.631377	-0.190315	-2.696285
H	-1.074633	-1.744344	0.886395
H	1.048060	-3.324617	0.095596
C	1.863155	-3.133500	-3.545200
H	0.893328	-2.765674	-3.881581
H	2.851949	-1.454985	-2.601478
H	1.741073	-4.188330	-3.284831
H	2.922896	-2.944479	-1.640468
C	2.887713	-2.981685	-4.689636
H	3.884913	-2.825823	-4.268580
H	2.937453	-3.922989	-5.244180
C	2.598534	-1.870969	-5.708184
H	1.709581	-2.099726	-6.299378
H	3.441331	-1.775617	-6.394042
S	2.321578	-0.193848	-5.058235
O	0.952852	-0.245799	-4.481716
O	3.336474	-0.002199	-4.013680
O	2.431283	0.681185	-6.205939
C	-2.776284	0.016472	-1.652383
H	-2.495780	-0.240190	-2.678060
H	-3.178382	-0.892481	-1.195432
C	-3.859466	1.101707	-1.667379
H	-4.123043	1.368054	-0.637742
H	-4.761034	0.664244	-2.103444
C	-3.487110	2.361269	-2.450726
H	-4.331628	3.051640	-2.492949
H	-2.652777	2.900839	-1.997619
H	-3.204630	2.118282	-3.477525

Table S7. List of coordinates obtained during geometry optimization for $C_6ImC_4SO_3$.

Atom	x, Å	y, Å	y, Å
C	0.445313	-0.845039	-1.737927
N	1.106968	-1.978728	-1.505965
N	-0.513144	-0.720540	-0.815669
C	-1.495297	0.371183	-0.779112
C	-0.460845	-1.811390	0.030335
C	0.557876	-2.598254	-0.405013
C	2.235042	-2.437745	-2.341923
H	-1.686223	0.604440	0.269891
H	-1.015383	1.237279	-1.230585
H	0.651895	-0.206194	-2.607401
H	-1.132131	-1.926948	0.862229
H	0.940397	-3.525648	-0.018931
C	1.787840	-3.078737	-3.667171
H	0.849157	-2.622369	-3.983357
H	2.846028	-1.557430	-2.549195

H	1.598536	-4.144179	-3.512487
H	2.812671	-3.134743	-1.734267
C	2.846469	-2.877967	-4.772292
H	3.839524	-2.814313	-4.318660
H	2.862443	-3.765954	-5.410730
C	2.638426	-1.665238	-5.689054
H	1.753532	-1.793486	-6.315529
H	3.501492	-1.551046	-6.346214
S	2.428518	-0.042081	-4.892478
O	1.041384	-0.072071	-4.359967
O	3.418928	-0.001862	-3.808357
O	2.618963	0.927340	-5.950279
C	-2.790544	0.011076	-1.506992
H	-2.551256	-0.236975	-2.545136
H	-3.208642	-0.892301	-1.053230
C	-3.836930	1.131019	-1.469056
H	-4.045868	1.402194	-0.427339
H	-4.772858	0.725791	-1.864719
C	-3.468677	2.385371	-2.265437
H	-2.573381	2.855961	-1.845349
H	-3.205553	2.097902	-3.289442
C	-4.594665	3.420283	-2.305508
H	-5.491064	2.956259	-2.730736
H	-4.859178	3.706753	-1.281640
C	-4.235836	4.668254	-3.109080
H	-5.061663	5.382852	-3.122338
H	-3.364994	5.174478	-2.684962
H	-3.997259	4.416622	-4.145172

Table S8. List of coordinates obtained during geometry optimization for $C_8ImC_4SO_3$.

Atom	x, Å	y, Å	y, Å
C	0.428274	-0.849342	-1.744586
N	1.073350	-1.991140	-1.505959
N	-0.556556	-0.726829	-0.850361
C	-1.529565	0.373401	-0.829562
C	-0.538814	-1.827615	-0.015791
C	0.486042	-2.618318	-0.429315
C	2.221839	-2.450463	-2.313433
H	-1.732533	0.611673	0.216133
H	-1.035879	1.233679	-1.277182
H	0.666969	-0.202956	-2.599741
H	-1.235850	-1.946928	0.794187
H	0.849019	-3.553257	-0.042523
C	1.808230	-3.087756	-3.650904
H	0.881925	-2.625164	-3.993583
H	2.840241	-1.571121	-2.502370
H	1.608031	-4.152163	-3.502659

H	2.782475	-3.149828	-1.692593
C	2.899104	-2.893191	-4.725262
H	3.879887	-2.841751	-4.244185
H	2.923422	-3.778557	-5.367084
C	2.728642	-1.674483	-5.641601
H	1.858350	-1.789489	-6.290786
H	3.609304	-1.567762	-6.276327
S	2.518411	-0.052402	-4.843062
O	1.119962	-0.070424	-4.340562
O	3.486059	-0.027424	-3.738187
O	2.741117	0.919270	-5.892509
C	-2.818325	0.022940	-1.573338
H	-2.567856	-0.231725	-2.607267
H	-3.251511	-0.874455	-1.121749
C	-3.853964	1.153358	-1.554188
H	-4.075976	1.429557	-0.516578
H	-4.787577	0.756114	-1.963121
C	-3.460375	2.401487	-2.348406
H	-2.568994	2.865531	-1.913327
H	-3.180455	2.106489	-3.365709
C	-4.576656	3.446152	-2.414627
H	-5.465922	2.984424	-2.854941
H	-4.859027	3.740716	-1.396434
C	-4.185920	4.693848	-3.208884
H	-3.275301	5.116911	-2.771794
H	-3.921246	4.402538	-4.231882
C	-5.261169	5.785714	-3.259366
H	-4.823749	6.681157	-3.710460
H	-5.537140	6.067378	-2.236948
C	-6.519118	5.404082	-4.040512
H	-7.219114	6.241393	-4.088899
H	-6.272484	5.120543	-5.067294
H	-7.046297	4.564560	-3.582623

The Bader analysis was performed using the Multiwfn program [S4], where Van der Waals volumes were obtained. The results of volume calculations are presented in Table S9.

Table S9. Van der Waals volumes of cations $[C_nC_4Im]^+$ anion $[CH_3SO_3]^-$ and ZILs $C_nImC_4SO_3$ (n = 4, 6, 8).

**Species**	**V, Å^3**
$[C_4C_4Im]^+$	272.8
$[C_4C_6Im]^+$	319.6
$[C_4C8_4Im]^+$	366.0
$[CH_3SO_3]^-$	102.5
$C_4ImC_4SO_3$	325.88

$C_6ImC_4SO_3$	372.63
$C_8ImC_4SO_3$	419.1

EPR of stochastic molecular librations

Stochastic librations or small-angle wobblings are limited stochastic orientation motions with small amplitudes that occur on a nanosecond time scale.

Earlier, in a series of works by Dzuba et.al.[S5,S6], a powerful approach for studying stochastic librational movements in organic glasses and biopolymers using pulse EPR by applying the nitroxide probes was developed. This method is based on the measurements of transverse relaxation times (T_2) of nitroxide in two definite spectral positions.

The theoretical consideration of spin relaxation induced by the fast (sub-microsecond) stochastic molecular librations predicts the exponential decay of the two-pulse electron spin echo (ESE) signal in the Hahn consequence upon incrementing the time delay between two pulses. The decay rate is determined with spectral anisotropy at the position of the nitroxide spectrum (positions I and II in Fig.S1a), the central component (I) is influenced mainly by the anisotropy of the g-tensor, with the anisotropy of HFI being negligible; therefore, it possesses the smallest anisotropy and the narrowest linewidth. For the broadest high-field component (II), both the anisotropies influence the spectral shape in an additive way, so it is the most anisotropic and the broadest one. Therefore, the decay rates for the field positions (I) and (II) are essentially different. However, there are other additive relaxation mechanisms contributing to the resulting T_2 values. Therefore, in order to elucidate pure libration-induced relaxation, it is necessary to subtract the relaxation rates (inverse relaxation times T_2) in the two spectral positions (I) and (II), as is illustrated in Fig. 1a.

As a result, we obtain a pure libration-based contribution, which we denoted $L \equiv (1/T_2(II) - 1/T_2(I))$. Redfield relaxation theory shows that for fast (sub-microsecond) and small-angle librations $L \approx C < \alpha^2 > \tau_c$, where $< \alpha^2 >$ is the mean square angular amplitude of motion, τ_C is the corresponding correlation time, and C is the numerical coefficient, whose value was semi-empirically determined to be $9*10^{-16}$ s^{-2} for nitroxide radicals[S6].

Measuring the values of the librational parameter at different temperatures, it is possible to plot a curve $L(T)$ the shape of which is more informative than the absolute value of L at a certain temperature. Theory of atomic displacements predicts that $L(T)$ should linearly grow with temperature. Figure S1b sketches possible behaviors of $L(T)$ dependence. The onset of librations indicates the temperature where librations start to

influence electron spin relaxation strong enough to be detected in ESE-based T_2 measurements. The slope of the $L(T)$ curve characterizes the intensity of librations. When glass softens and transforms into a liquid, the amplitude of the nitroxide motions drastically grows, leading to a steep rise of $L(T)$ until T_2 becomes too short to be measured. Thus, in general, no other behaviors are to be expected for the $L(T)$, unless some structural rearrangements occur. There is no reason for the $L(T)$ curve to change from a rising trend to a decrease as T increases, because kT driving librations does grow. Thus, any deviations from monotonic linear growth indicate some structural changes in the glassy matrix surrounding the nitroxide.

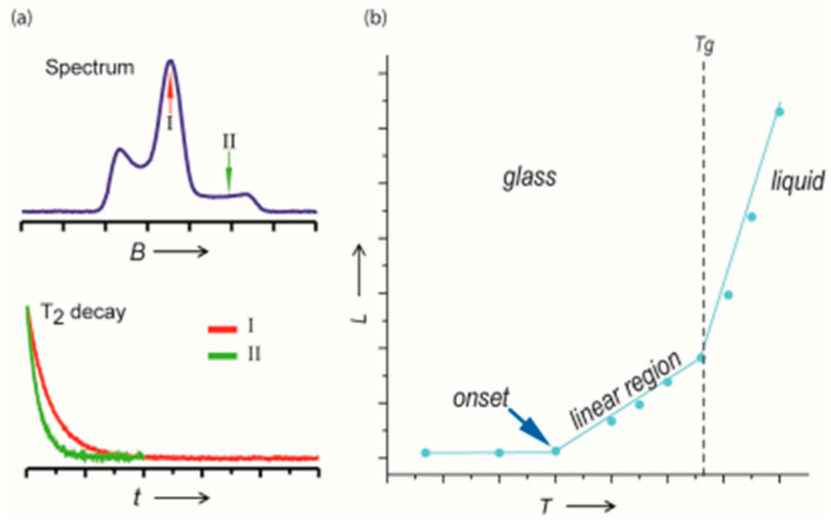

Figure S1. Sketch of the pulse EPR analysis scheme. (a) Two-pulse electron spin-echo-detected spectrum (top). The T_2 decay is measured by incrementing delay in $\pi/2$ - τ - π - τ - echo sequence at two spectral positions I and II. Corresponding T_2 times are obtained with monoexponential analysis. (b) Typical $L(T)$ dependence showing low-temperature region of no librations, then their onset, linear region of effective librations, and, finally, transition into a liquid state with steep increase of molecular motion.

However, in the previously studied ILs, the $L(T)$ curve has three characteristic regions that are marked as {a}, {b}, and {c} in Figure S2. The increase in the $L(T)$ function at ~70 K (region {a}) indicates the onset of stochastic molecular librations in IL. Further increase of the stochastic librations amplitude [growth of $L(T)$] is observed up to ~140 K (region {a} in Fig.S2). However, then the anomalous suppression of the stochastic librations is found within ~140–200 K (region {b}), which has never been observed in common organic glasses or biological membranes. At even higher temperatures $T > 200$ K, which are close to T_g of the studied ILs, the trend reverts to increase again (region {c}), to be assigned to the unlocking of diffusive rotation of the radical in softened/melted IL. The linear growth of $L(T)$ in region {a} is a typical behavior that was observed previously in various organic glasses and biological membranes. The

nonlinear region {b} of *L(T)* is the most interesting and represents the structural rearrangements in bulk IL. It was noticed that the position of local minimum of *L(T)* curve clearly coincides with the T_g temperature of given IL.

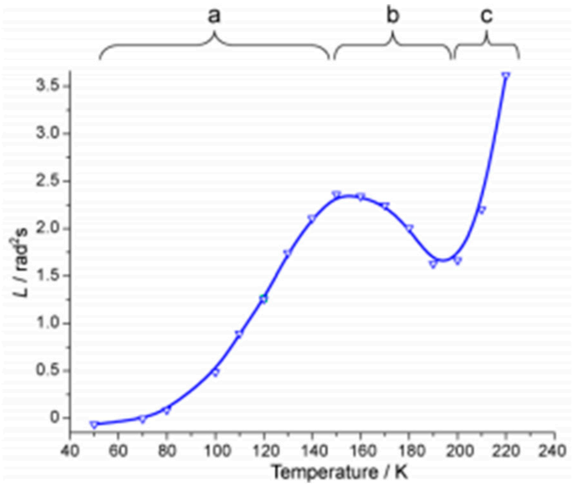

Figure S2. Representative schematic temperature dependence of the motional parameter *L* for nitroxide radical in ILs {a}, {b}, and {c} indicate the motional regimes, see text for details. {b} is the region of anomaly.

Continuous wave EPR

Tables S10-S15 demonstrate the spectroscopic parameters used in CW EPR spectra simulations of spin probe TEMPO-D_{18} dissolved in the corresponding glass-formers. The computer simulations were performed using EasySpin[S7].

Table S10. List of parameters used in simulation of CW EPR spectra of TEMPO-D18 in $[C_4C_4Im][CH_3SO_3]$.

		Mobile fraction			Immobile fraction		
T / K	M	τc / ns	g-tensor	A-tensor / MHz	g-tensor	A-tensor / MHz	Astrain / MHz
150	0	-	[2.0117 2.0081 2.0041]	[17.5 19.7 96.5]	[2.0117 2.0081 2.0041]	[17.5 19.7 96.5]	[0 0 5.9]
160	0	-					
170	0	-					
180	0	-					
190	0.10	5.8		[17.5 19.7 95.2]		[17.5 19.7 95.2]	
200	0.17	8.2					
210	0.32	8.4					
220	0.49	8.5					
230	0.72	8.4					
240	0.96	6.3		[17.5 19.7 100.0]		[17.5 19.7 100.0]	
250	0.98	4.2		[17.5 19.7 103.5]		[17.5 19.7 103.5]	

260	1	3.0		[17.5 19.7 102.5]		[17.5 19.7 102.5]	
270	1	2.0		[17.5 19.7 99.0]		[17.5 19.7 99.0]	

Table S11. List of parameters used in simulation of CW EPR spectra of TEMPO-D18 in $[C_6C_4Im][CH_3SO_3]$.

		Mobile fraction			Immobile fraction		
T / K	M	τc / ns	g-tensor	A-tensor / MHz	g-tensor	A-tensor / MHz	Astrain / MHz
140	0	-	[2.0110 2.0074 2.0034]	[17.5 19.6 96.7]	[2.0110 2.0074 2.0034]	[17.5 19.6 96.7]	[0.7 0 6.4]
150	0	-					
160	0	-					
170	0	-					
180	0,15	5,8					
190	0,31	6,7					
200	0,52	8,3					
210	0,71	7,6					
220	0,93	5,7		[17.5 19.6 102.2]		[17.5 19.6 102.2]	
230	1	3,8		[17.5 19.6 105.2]		[17.5 19.6 105.2]	
240	1	2,9		[17.5 19.6 101.5]		[17.5 19.6 101.5]	
250	1	2,2		[17.5 19.6 100.0]		[17.5 19.6 100.0]	

Table S12. List of parameters used in simulation of CW EPR spectra of TEMPO-D18 in $[C_8C_4Im][CH_3SO_3]$.

		Mobile fraction			Immobile fraction		
T / K	M	τc / ns	g-tensor	A-tensor / MHz	g-tensor	A-tensor / MHz	Astrain / MHz
140	0	-	[2.0116 2.0079 2.0039]	[17.4 19.7 95.8]	[2.0116 2.0079 2.0039]	[17.4 19.7 95.8]	[0 0 7.0]
150	0	-					
160	0	-					
170	0,07	4,5					
180	0,13	4,6					
190	0,24	6.0					
200	0,38	6,7					
210	0,59	7,3					
220	0,75	6,8					
230	0,93	4,8		[17.4 19.7		[17.4 19.7	

				103.4]		103.4]	
240	0,98	3,9		[17.4 19.7 105.2]		[17.4 19.7 105.2]	
250	1	3,1		[17.4 19.7 103.5]		[17.4 19.7 103.5]	
260	1	2,5		[17.4 19.7 102.6]		[17.4 19.7 102.6]	

Table S13. List of parameters used in simulation of CW EPR spectra of TEMPO-D18 in $C_4ImC_4SO_3$.

		Mobile fraction			Immobile fraction		
T / K	M	τc / ns	g-tensor	A-tensor / MHz	g-tensor	A-tensor / MHz	Astrain / MHz
140	0	-	[2.0108 2.0072 2.0032]	[18.0 19.5 97.1]	[2.0108 2.0072 2.0032]	[18.0 19.5 97.1]	[4.4 0 6.2]
150	0	-					
160	0	-					
170	0	-					
180	0	-					
190	0	7,8					
200	0,15	10,1		[18.0 19.5 96.3]		[18.0 19.5 96.3]	
210	0,23	9,7					
220	0,31	8,8					
230	0,42	8,5					
240	0,50	9,3					
250	0,62	8,9					
260	0,71	9,1					
270	0,87	8,6		[18.0 19.5 98.4]		[18.0 19.5 98.4]	
280	0,98	7,4		[18.0 19.5 99.7]		[18.0 19.5 99.7]	
290	1	5,6		[18.0 19.5 100.8]		[18.0 19.5 100.8]	
300	1	4,4		[18.0 19.5 103.5]		[18.0 19.5 103.5]	
310	1	7,8		[18.0 19.5 105.9]		[18.0 19.5 105.9]	

Table S14. List of parameters used in simulation of CW EPR spectra of TEMPO-D18 in $C_6ImC_4SO_3$.

		Mobile fraction			Immobile fraction		
T / K	M	τc / ns	g-tensor	A-tensor / MHz	g-tensor	A-tensor / MHz	Astrain / MHz

140	0	-	[2.0108 2.0072 2.0032]	[18.0 19.5 97.1]	[2.0108 2.0072 2.0032]	[18.0 19.5 97.1]	[4.7 0 6.5]
150	0	-					
160	0	-					
170	0	-					
180	0	-					
190	0,11	6,2		[18.0 19.5 96.4]		[18.0 19.5 96.4]	
200	0,22	8,6					
210	0,33	10,4					
220	0,43	9,7					
230	0,53	9,7					
240	0,62	9,6					
250	0,72	9,1					
260	0,87	8,3		[18.0 19.5 98.8]		[18.0 19.5 98.8]	
270	1	6,9		[18.0 19.5 102.4]		[18.0 19.5 102.4]	
280	1	5,3		[18.0 19.5 105.3]		[18.0 19.5 105.3]	
290	1	4,4		[18.0 19.5 103.9]		[18.0 19.5 103.9]	
300	1	3,8		[18.0 19.5 104.3]		[18.0 19.5 104.3]	

Table S15. List of parameters used in simulation of CW EPR spectra of TEMPO-D18 in $C_8ImC_4SO_3$.

		Mobile fraction			Immobile fraction		
T / K	M	τc / ns	g-tensor	A-tensor / MHz	g-tensor	A-tensor / MHz	Astrain / MHz
140	0	-	[2.0109 2.0072 2.0032]	[18.0 19.5 97.2]	[2.0109 2.0072 2.0032]	[18.0 19.5 97.2]	[4.4 0 6.1]
150	0	-					
160	0	-					
170	0	-					
180	0	-					
190	0,14	5,9		[18.0 19.5 96.3]		[18.0 19.5 96.3]	
200	0,24	8,2					
210	0,35	9.0					
220	0,48	9,7					
230	0,57	7,7					
240	0,67	7,5					
250	0,82	7,7		[18.0 19.5 97.9]		[18.0 19.5 97.9]	
260	0,97	6,5		[18.0 19.5 102.3]		[18.0 19.5 102.3]	
270	0,99	4,8		[18.0 19.5		[18.0 19.5	

				106.0]		106.0]	
280	1	4,0		[18.0 19.5 104.9]		[18.0 19.5 104.9]	
290	1	3,4		[18.0 19.5 103.0]		[18.0 19.5 103.0]	
300	1	3,0		[18.0 19.5 102.3]		[18.0 19.5 102.3]	

Figures S3, S4 show the CW EPR spectra of spin probe TEMPO-D18 dissolved in the corresponding samples.

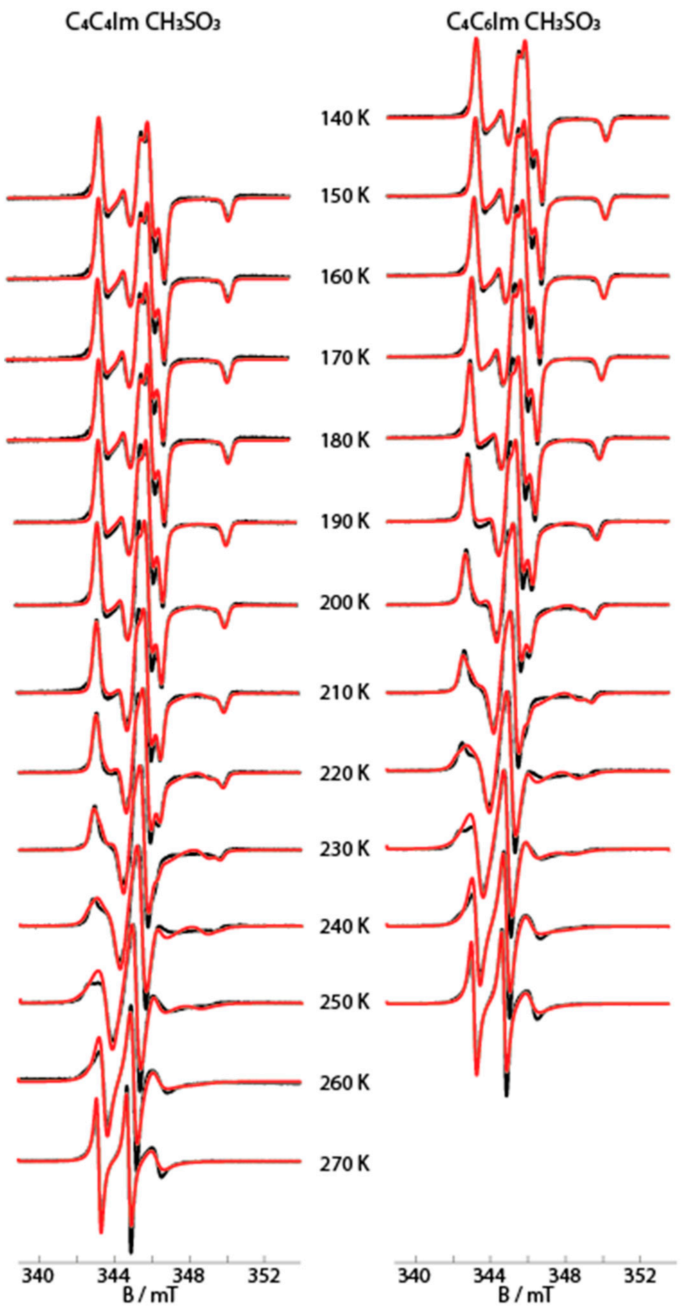

Figure S3. CW EPR spectra of TEMPO-D18 dissolved in the corresponding IL vs. temperature. Black lines represent the experimental data, red ones - the simulation results.

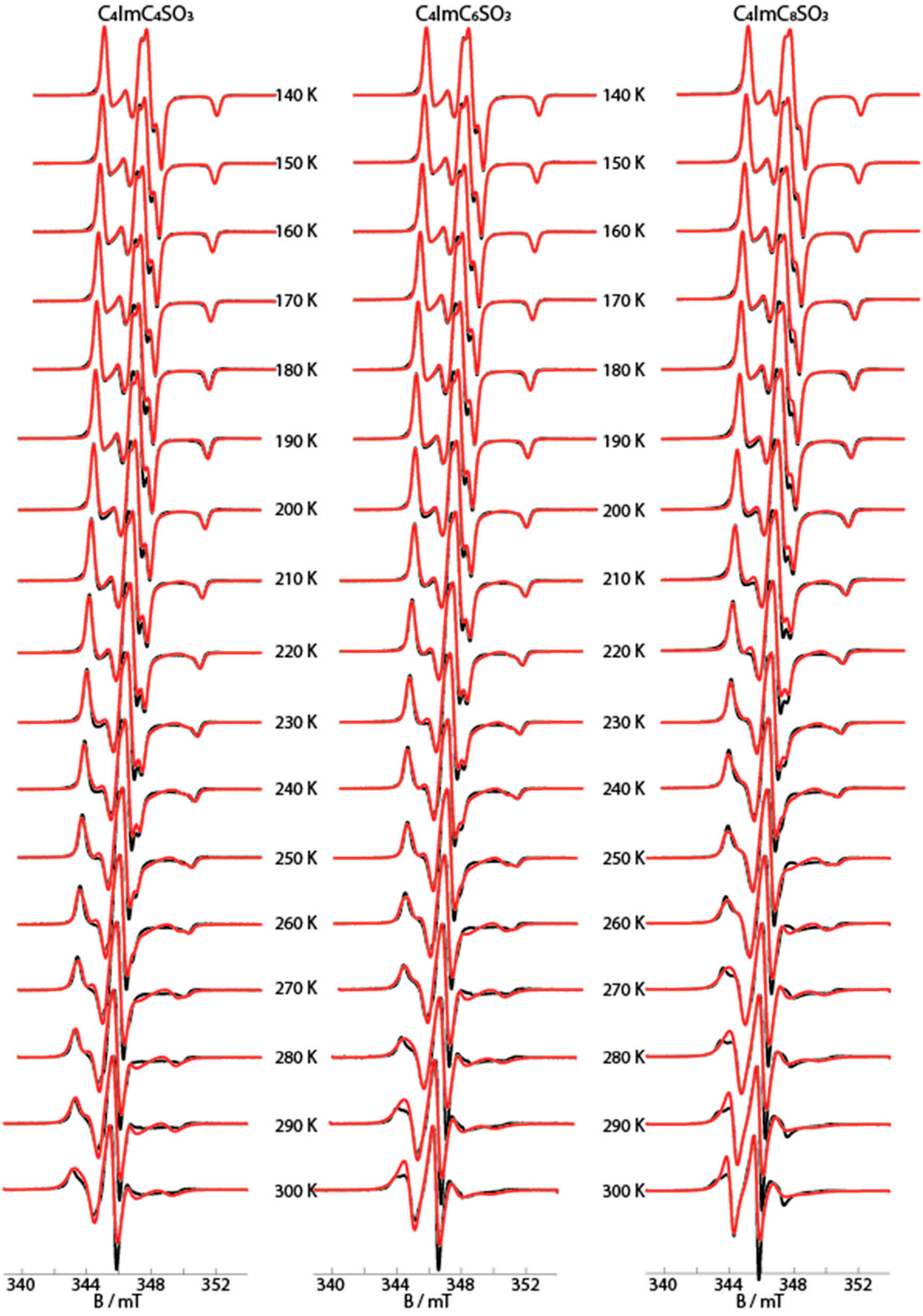

Figure S4. CW EPR spectra of TEMPO-D18 dissolved in the corresponding ZIL vs. temperature. Black lines represent the experimental data, red ones - the simulation results.

References

S1. Cai, J.; Liu, J.; Mu, S.; Liu, J.; Hong, J.; Zhou, X.; Ma, Q.; Shi, L. Corrosion inhibition effect of three imidazolium ionic liquids on carbon steel in chloride contaminated environment. *Int. J. Electrochem. Sci.* **2020**, *15*, 1287–1301, doi:10.20964/2020.02.12.

S2. Dupont, J.; Consorti, C.S.; Suarez, P. a. Z.; De Souza, R.F. Preparation of 1-butyl-3-methyl imidszolium-based room temperature ionic liquids. *Org. Synth.* **2002**, *79*, 236, doi:10.15227/orgsyn.079.0236.

S3. Neese, F. The ORCA program system. *Wiley Interdiscip. Rev. Comput. Mol. Sci.* **2012**, *2*, 73–78, doi:10.1002/wcms.81.

S4. Lu, T.; Chen, F. Multiwfn: A multifunctional wavefunction analyzer. *J. Comput. Chem.* **2012**, *33*, 580–592, doi:10.1002/jcc.22885.

S5. Dzuba, S.A. Libration motion of guest spin probe molecules in organic glasses : CW EPR and electron spin echo study. *Spectrochim. Acta - Part A Mol. Biomol. Spectrosc.* **2000**, *56*, 227–234.

S6. Isaev, N.P.; Dzuba, S.A. Fast Stochastic Librations and Slow Rotations of Spin Labeled Stearic Acids in a Model Phospholipid Bilayer at Cryogenic Temperatures. *J. Phys. Chem. B* **2008**, *112*, 13285–13291.

S7. Stoll, S.; Schweiger, A. EasySpin, a comprehensive software package for spectral simulation and analysis in EPR. *J. Magn. Reson.* **2006**, *178*, 42–55, doi:10.1016/j.jmr.2005.08.013.
